# Estimation of cardiac output and pulmonary vascular resistance by contrast echocardiography transit time measurement: a prospective pilot study

**DOI:** 10.1186/1476-7120-12-44

**Published:** 2014-10-31

**Authors:** Brian G Choi, Reza Sanai, Benjamin Yang, Heather A Young, Ramesh Mazhari, Jonathan S Reiner, Jannet F Lewis

**Affiliations:** The GW Heart & Vascular Institute, The George Washington University, Washington, DC USA; Department of Epidemiology & Biostatistics, The George Washington University, Washington, DC USA

**Keywords:** Contrast echocardiography, Hemodynamics, Cardiac output, Pulmonary vascular resistance

## Abstract

**Background:**

Studies with other imaging modalities have demonstrated a relationship between contrast transit and cardiac output (CO) and pulmonary vascular resistance (PVR). We tested the hypothesis that the transit time during contrast echocardiography could accurately estimate both CO and PVR compared to right heart catheterization (RHC).

**Methods:**

27 patients scheduled for RHC had 2D-echocardiogram immediately prior to RHC. 3 ml of DEFINITY contrast followed by a 10 ml saline flush was injected, and a multi-cycle echo clip was acquired from the beginning of injection to opacification of the left ventricle. 2D-echo based calculations of CO and PVR along with the DEFINITY-based transit time calculations were subsequently correlated with the RHC-determined CO and PVR.

**Results:**

The transit time from full opacification of the right ventricle to full opacification of the left ventricle inversely correlated with CO (r = -0.61, p < 0.001). The transit time from peak opacification of the right ventricle to first appearance in the left ventricle moderately correlated with PVR (r = 0.46, p < 0.01). Previously described echocardiographic methods for the determination of CO (Huntsman method) and PVR (Abbas and Haddad methods) did not correlate with RHC-determined values (p = 0.20 for CO, p = 0.18 and p = 0.22 for PVR, respectively). The contrast transit time method demonstrated reliable intra- (p < 0.0001) and inter-observer correlation (p < 0.001).

**Conclusions:**

We describe a novel method for the quantification of CO and estimation of PVR using contrast echocardiography transit time. This technique adds to the methodologies used for noninvasive hemodynamic assessment, but requires further validation to determine overall applicability.

## Background

Invasive hemodynamic assessment by right heart catheterization (RHC) is a mainstay of evaluation of patients with pulmonary hypertension and congestive heart failure, [1, 2] but this procedure subjects patients to risks including venous access complications, arrhythmias and, in rare circumstances, even death [3]. Non-invasive hemodynamic assessment has become commonplace, often supplanting invasive assessment. Several echocardiographic methods have been developed using echocardiography to estimate pulmonary vascular resistance (PVR) and cardiac output (CO) [4–8]. However, accurate assessment of right heart pressures and pulmonary vascular resistance, compared to invasive measurements has proven less reliable. One different approach was utilized by Galanti *et al.* [9], who measured transpulmonary transit times of intravenous Albunex in dogs as an indicator of cardiac output. They noted an excellent correlation between the pulmonary transit rate, as measured by the time to first echocardiographic presence in the left ventricle, with thermodilution cardiac output. This technique, however, has not been validated in humans.

Ultrasonic contrast agents have been used to improve image quality for echocardiography, [10] but a potential role in assessment of PVR and CO has not yet been defined. Investigators using other imaging modalities have suggested using transit times to assess these measures [11–13]. In this prospective pilot study, we tested the hypothesis that transit time assessment during contrast-enhanced echocardiography could accurately estimate both PVR and CO compared to the gold-standard of RHC in patients without evidence of structural right heart disease.

## Methods

### Patients

38 consecutive adult patients clinically referred for right-heart catheterization were evaluated for potential inclusion in the study. The exclusion criteria were known or suspected right-to-left, bi-directional, or transient right-to-left cardiac shunts or pulmonary arteriovenous malformations (AVM), tamponade, previously documented moderate to severe tricuspid or pulmonic insufficiency, right ventricular hypokinesis, or prior adverse reaction to Definity or hypersensitivity to perflutren. The study was approved by George Washington University institutional review board, and informed, written consent was obtained from all patients. After consent, 5 patients were found to not meet enrollment criteria: 4 did not have right-heart catheterization (including 1 with suspected tamponade), 1 had right ventricular systolic dysfunction. 6 patients were excluded from analysis secondary to timing errors with contrast injection (i.e., images were not acquired simultaneously with contrast injection, the onset of intracardiac contrast arrival was not acquired, the peak opacification in the left ventricle was not acquired). The remaining 27 patients were included in the final analysis.

### Contrast echo protocol

Immediately prior to RHC, a complete 2D transthoracic echocardiogram with Doppler using a full platform echocardiographic instrument (Philips iE33; Andover, MA) was performed on each patient in the pre-procedural holding area) [14]. Prior to the conclusion of the study, 3 mL diluted perflutren lipid microsphere solution (8.7 mL normal saline plus 1.3 mL of activated Definity, Lantheus Medical Imaging, N. Billerica, MA, USA) was injected through an 18-Gauge right antecubital intravenous catheter followed by 10 mL normal saline push, with a multi-cycle echo clip in the apical 4-chamber window started with the beginning of the injection.

### Echocardiographic hemodynamic assessment

PVR was calculated using two methods: (1) the Abbas *et al.* method [4] which uses the ratio of peak tricuspid regurgitant velocity to right ventricular outflow tract time-velocity integral (TVI_RVOT_), and (2) the Haddad *et al.* method [5] which uses the ratio of the systolic pulmonary artery pressure to heart rate (PASP/HR) × TVI_RVOT_. The CO was calculated by the Huntsman *et al.* method, [8] which uses heart rate × stroke volume, where stroke volume is calculated as TVI_LVOT_ × LVOT cross-sectional area.

### Right heart catheterization

RHC was performed using a Swan-Ganz catheter introduced via femoral, internal jugular or brachial vein approach following standard methods. CO was calculated using Fick principle with measured oxygen consumption, and PVR was calculated using the formula: 80 (mean pulmonary artery pressure – pulmonary capillary wedge pressure)/CO.

### Image analysis

**Figure 1:**
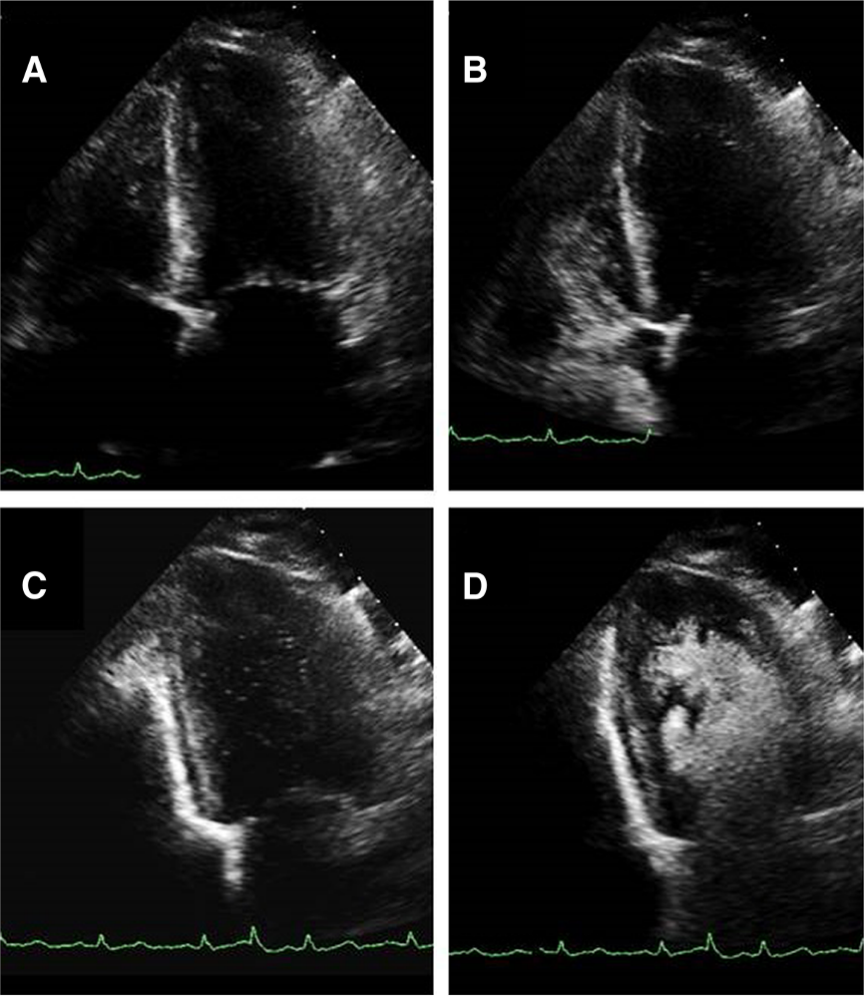
**Representative apical 4-chamber view images demonstrating (A) left ventricle and right ventricle prior to contrast injection, (B) full opacification by Definity contrast of the right ventricle, (C) first appearance of contrast in the left ventricle, and (D) full opacification of the left ventricle.**

The multi-cycle echo clip of the contrast injection was evaluated frame-by-frame using IMPAX® Cardiovascular Review Station version 7.8 (Agfa HealthCare N.V.; Morstel, Belgium) to identify time to first bubble appearance in the right ventricle (RV1), full opacification of the right ventricle (RVFull), peak opacification of the right ventricle (RVPeak), first bubble in the left ventricle (LV1), full opacification of the left ventricle (LVFull), and peak opacification of the left ventricle (LVPeak). For example, in a 26-Hertz clip, 520 frames are recorded in 20 seconds, and the clip was played back frame-by-frame to determine the frame in which RV1, RVFull, RVPeak, LV1, LVFull, and LVPeak occur, and then the frame number would be divided by 26 to determine the time in seconds. Full opacification was defined as the moment the contrast has filled the entire ventricle; peak opacification was defined as the moment that contrast brightness has subjectively reached its peak within the ventricle. Representative images are shown in Figure 1. The measurements were each read twice by two independent readers, with one month between each series of readings, and all readers were blinded to previous measurements.

### Statistical analysis

The reference standards for CO and PVR were determined by RHC, and were correlated to the established echo methods for determination of CO [8] and PVR [4, 5] by Pearson’s correlation. CO and PVR were then correlated to contrast transit time intervals to determine the interval with best correlation. Once the transit time intervals that best correlated to CO and PVR were established, these intervals were re-measured by the same reader one month later to establish intra-observer variability and by a second independent reader for inter-observer variability as determined by Spearman correlation. Statistical analysis used SAS version 9.3 (Cary, NC). A p value less than 0.05 was considered statistically significant.

## Results

27 patients were included in this analysis (Table 1). The average age was 60 ± 13 years, 52 percent were female, and the most common indication for RHC was for evaluation of suspected pulmonary hypertension. As calculated by RHC, the mean cardiac output was 6.2 ± 1.4 L/min; cardiac index was 3.1 ± 0.8 L/min/m^2^. The mean PVR was 1.8 ± 1.2 Wood units (WU).

**Table 1:** **Clinical characteristics of study patients**

Characteristics	
**Mean Age ± SD**	**60 ± 12.8 years**
**Females (%)**	**52%**
**Body Surface Area ± SD**	**1.98 ± 0.21 m** ^**2**^
**Indications for RHC**	
**Pulmonary Hypertension**	**9 (33%)**
**Dyspnea**	**8 (30%)**
**Cardiomyopathy**	**7 (26%)**
**Acute Heart Failure**	**3 (11%)**
**LV EF (%)**	**50 ± 16.0**
**Mean RA pressure (mm Hg)**	**8 ± 5**
**Mean PA pressure (mm Hg)**	**26 ± 10.7**
**Mean PCWP (mm Hg)**	**14 ± 7.7**
**Cardiac Output (L/min)**	**6.15 ± 1.4**
**Cardiac Index (L/min/m** ^**2**^ **)**	**3.06 ± 0.77**
**PVR (WU)**	**1.78 ± 1.15**
**PVR (WU/m** ^**2**^ **)**	**0.88 ± 0.56**

**Figure 2:**
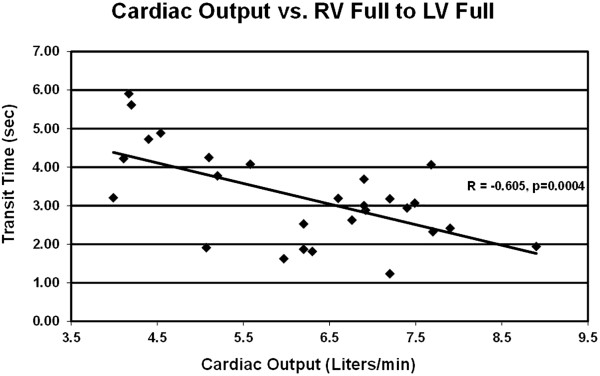
**Cardiac output by right heart catheterization correlated to Definity contrast transit time between full opacification of the right ventricle and full opacification of the left ventricle.**

**Figure 3:**
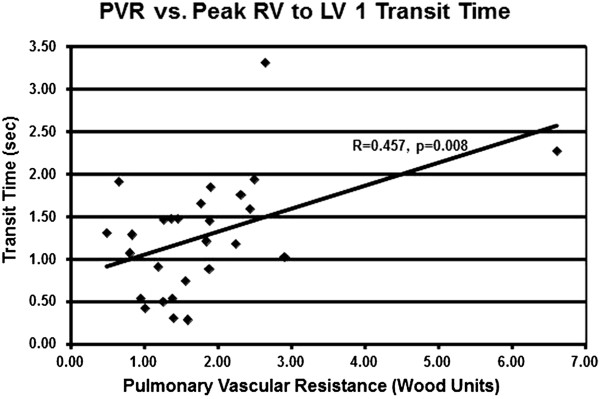
**Pulmonary vascular resistance by right heart catheterization correlated to Definity contrast transit time between peak opacification of the right ventricle and first appearance in the left ventricle.**

CO determined by echo, using the Huntsman method, was 4.3 ± 1.5 L/min. Correlation of echo CO by Huntsman method and RHC CO by Fick method showed no significant correlation (r = 0.15, p = 0.20). Echo calculation of PVR, using either the Abbas or the Haddad method, showed no significant correlations to PVR determined by RHC (Table 2).Using echo contrast transit times, CO best correlated to the time between RVFull and LVFull (r = -0.61, p < 0.001), with shorter transit time correlating to higher CO (Figure 2). This mean transit time was 3.2 ± 1.2 seconds. From the linear regression analysis, CO =13 – (2 × transit time). Intra-observer Spearman correlation was 0.92 (p < 0.0001), and inter-observer Spearman correlation was 0.79 (p < 0.001). The mean intra-observer difference was 0.4 ± 0.3 seconds; the mean inter-observer difference was 2.1 ± 1.5 seconds.PVR best correlated to the time between RVPeak and LV1 (r = 0.46, p < 0.01), with a longer transit time correlating with higher PVR (Figure 3). The mean transit time was 1.3 ± 0.7 seconds. From the linear regression analysis, PVR = (3.7 × transit time) – 2.9. Intra-observer Spearman correlation was 0.82 (p < 0.0001), and inter-observer Spearman correlation was 0.79 (p < 0.001). The mean intra-observer difference was 0.4 ± 0.3 seconds; the mean inter-observer difference was 1.0 ± 0.7 seconds.

**Table 2:** **Correlation of cardiac output (CO) and pulmonary vascular resistance (PVR) as determined by right heart catheterization (RHC) and established methods of echocardiographic determination** [4, 5, 8]

Method of calculation	RHC measured Mean ± SD	ECHO calculated Mean ± SD	Correlation	P value
**CO-Huntsman (L/min)**	**6.17 ± 1.37**	**4.34 ± 1.47**	**0.152**	**0.204**
**PVR-Abbas (TRV/RVOT × 10) Wood Units**	**1.44 ± 1.07**	**1.59 ± 0.71**	**0.182**	**0.181**
**PVR-Haddad (SPAP/(RVOT VTI×HR)) Wood Units/m** ^**2**^	**0.72 ± 0.52**	**0.26 ± 0.15**	**0.158**	**0.216**

## Discussion

Our results suggest that the estimation of PVR and CO using ultrasonic contrast agent transit times during echocardiography is feasible, accurate and reproducible. Using our methodology, PVR and CO correlated better with RHC-derived PVR and CO compared to previously established methods using echocardiography. Furthermore, our proposed method may be performed on any PACS system that allows frame-by-frame visualization. Slow transit from the right to left heart corresponded to decreased CO and increased PVR. Definity microsphere particles generally range in size of 1.1-3.3 microns with a maximum of 20 microns which facilitate its passage across the pulmonary vasculature (pulmonary capillary diameter averages 7 microns) into left heart chambers [15]. For this reason, we speculate that the reason PVR correlates with transit time is that a decrease in average pulmonary capillary diameter would slow transit; therefore, from peak contrast enhancement in the right ventricle to its first appearance in the left heart may correlate to PVR. The time the contrast takes to fully opacify the right ventricle to full opacification of the left ventricle would be more rapid with high cardiac output, which is what we found in the current study. In addition, our PVR method is derived independently of the need for the estimation of the pulmonary artery systolic pressure, which is not possible in cases where the tricuspid regurgitant jet is inadequate.

Previously established methods for estimation of PVR [4, 5] did not correlate with results found by cardiac catheterization. Notably, other studies also found poor correlation with these methods, especially for those patients with high PVR [16, 17], underscoring the need for alternative methods of non-invasive determination of PVR that may more reproducible. A limitation of the Abbas method [4] is its reliance on the velocity time integral of the right ventricular outflow tract, a measurement that is not easily measurable in the patient with significant pulmonary hypertension [18]. Furthermore, this method requires assessment of the tricuspid regurgitant jet velocity, a measure that may not be assessed for every patient if the jet is inadequate [19]. Similarly, the Haddad method [5] is also dependent on a reliably assessable transtricuspid regurgitant jet and right ventricular outflow tract velocity time integral. Our proposed method is independent of the need to assess these measures and may explain why our proposed method correlated better with catheterization-derived values.

Other investigators have pointed out the limitations of the Huntsman method [8] for determination of cardiac output by ultrasound: accurate velocity measurement requires good alignment between Doppler beam and blood flow [20] and reliable measurement of cross-sectional area for flow [21]. With the increased scrutiny of the echocardiographic measurement of left ventricular outflow tract dimensions, it is now well understood that the cross-section of the left ventricular outflow tract is not circular and relying on the diameter alone to calculate the cross-sectional area may lead the additional inaccuracy [22]. Our method is independent of these measurements and may explain the better correlation with CO.

### Limitations

In an effort to reduce confounding sources of variability, this pilot study was limited to patients without evidence of structural right heart disease. We presumed that severe tricuspid regurgitation, severe pulmonary regurgitation, and right ventricular systolic dysfunction would prolong transit times and PVR and CO would not correlate in these circumstances. In addition, 6 patients were excluded from analysis because of timing errors with contrast injection. Our sonographers were not previously accustomed to timing image acquisition with the moment of contrast injection, and our protocol required a higher level of coordination between the sonographer and the person injecting the contrast agent. We suspect that this coordination issue may be a reason that our derived measures that correlated best with PVR and CO are measures that are independent of the moment of initial injection. PVR requires timing from the moment of peak RV opacification and CO requires timing from the moment of RV full opacification; both of these moments are independent of the time of initial injection.

Definity contrast is not without safety limitations. The rates of serious adverse reactions associated with DEFINITY use are extremely rare as was demonstrated by retrospective cohort study of 42,408 patients, and 1 in 10,000 patients experienced an allergic reaction [23]. A prospective multicenter trial found no serious adverse events in 1,060 patients, but the Definity-related adverse event rate was 3.5%, primarily headache, nausea, back pain and tremor [24].

## Conclusions

This pilot study demonstrates that estimation of PVR and CO by measurement of ultrasonic contrast times may be superior to previously established echocardiographic methods. Further validation of this novel method may obviate the need for invasive assessment.
